# A rapid preparation procedure for laser microdissection-mediated harvest of plant tissues for gene expression analysis

**DOI:** 10.1186/s13007-019-0471-3

**Published:** 2019-08-02

**Authors:** Stian Olsen, Kirsten Krause

**Affiliations:** Department of Arctic and Marine Biology, Faculty of Biosciences, Fisheries and Economics, UiT The Arctic University of Norway, Framstredet 39, 9019 Tromsø, Norway

**Keywords:** *Cuscuta reflexa*, Gene expression, Laser microdissection, Parasitic plants, qPCR, Vibratome

## Abstract

**Background:**

Gene expression changes that govern essential biological processes can occur at the cell-specific level. To gain insight into such events, laser microdissection is applied to cut out specific cells or tissues from which RNA for gene expression analysis is isolated. However, the preparation of plant tissue sections for laser microdissection and subsequent RNA isolation usually involves fixation and embedding, processes that are often time-consuming and can lower the yield and quality of isolated RNA.

**Results:**

Infection sites of the parasitic plant *Cuscuta reflexa* growing on its compatible host plant *Pelargonium zonale* were sectioned using a vibratome and dried on glass slides at 4 °C before laser microdissection. High quality RNA (RQI > 7) was isolated from 1 mm^2^, 3 mm^2^ and 6 mm^2^ total surface areas of laser microdissection-harvested *C. reflexa* tissue, with the yield of RNA correlating to the amount of collected material (on average 7 ng total RNA/mm^2^). The expression levels of two parasite genes previously found to be highly expressed during host plant infection were shown to differ individually between specific regions of the infection site. By drying plant sections under low pressure to reduce the dehydration time, the induced expression of two wound-related genes during preparation was avoided.

**Conclusions:**

Plants can be prepared quickly and easily for laser microdissection by direct sectioning of fresh tissue followed by dehydration on glass slides. We show that RNA isolated from material treated in this manner maintains high quality and enables the investigation of differential gene expression at a high morphological resolution.

**Electronic supplementary material:**

The online version of this article (10.1186/s13007-019-0471-3) contains supplementary material, which is available to authorized users.

## Background

Currently, most research on how the dynamic expression of genes allows plants to control their own development and to respond to stimuli, requires the extraction and isolation of RNA prior to the actual gene expression analysis. Plant organs typically consist of multiple cell and tissue types, and there is an increasing awareness that essential gene expression changes occurring in specific cells or tissues can be overlooked when RNA is isolated from entire organs [1–3]. Laser microdissection (LM) combines microscopy and laser beams to isolate specific tissue or cell types from sections of a specimen [4, 5]. In plants, the technique has successfully been applied to study tissue- and cell-specific dynamics of a number of different biomolecules including carbohydrates, metabolites and proteins as well as RNA [6–8]. As the latter is prone to degradation by the activity of the almost omnipresent ribonucleases, the usage of LM to harvest material for gene expression analysis requires a fine-tuned balance between maintaining both histological detail and RNA quality. The crucial stage at which the fate of both tissue morphology and RNA integrity is decided, is the initial preparation of tissues prior to the actual LM. Traditionally, such preparations are time-consuming and involve fixation of the specimen followed by embedding in a compound to allow sectioning of thin slices that are subsequently used for LM [9, 10]. Paraffin sectioning and cryosectioning are two frequently used methods to prepare sections for LM. In general, RNA isolated from cryosectioned samples has higher quality, whereas paraffin embedding is preferred when the preservation of histological integrity for the identification of specific cell types is essential [11, 12]. Sample fixation is performed to maintain tissue morphology and to suspend the status of biomolecules during the processes of embedding, sectioning and slide preparation. However, the use of fixatives has been shown to reduce the quality and/or extractability of RNA [13, 14]. Gotte et al. [15] demonstrated that gene expression analysis can be performed on LM-harvested plant material without prior fixation, albeit tissues were not sectioned but directly laser microdissected.

Here, we present a rapid method for preparing plant tissue sections for LM-mediated harvest of material for RNA isolation and apply it to study region-specific gene expression patterns of the infection organ of the parasitic plant *Cuscuta reflexa*. Parasitic plants steal resources from other plants by developing specialized infection organs called haustoria that invade the tissue of host plants and establish vascular connections between the parasite and its host [16, 17]. LM is an ideal method to unravel molecular changes at the immediate interface where cells from both plants meet [18]. Our method involves vibratome sectioning of freshly harvested infection sites of *C. reflexa* on its compatible host plant *Pelargonium zonale* and subsequent dehydration of sections on glass slides. The preparation procedure was evaluated by collecting different amounts of tissue for RNA isolation and comparing RNA yield and quality. Subsequently, the applicability of the method was demonstrated by quantifying the differential expression of the previously identified haustorium-related genes, *Cuscuta reflexa* xyloglucan endotransglucosylase/hydrolase-1 (*Cr*-*XTH*-*1*) and *Cuscuta reflexa* peroxidase-2 (*Cr*-*PX*-*2*) [19], in specific regions of the *C. reflexa* infection organ. To address the inevitable induction of stress gene expression when sectioning live plant material, the expression levels of two wound-related genes were quantified under different sample treatments.

## Results

**Fig. 1:**
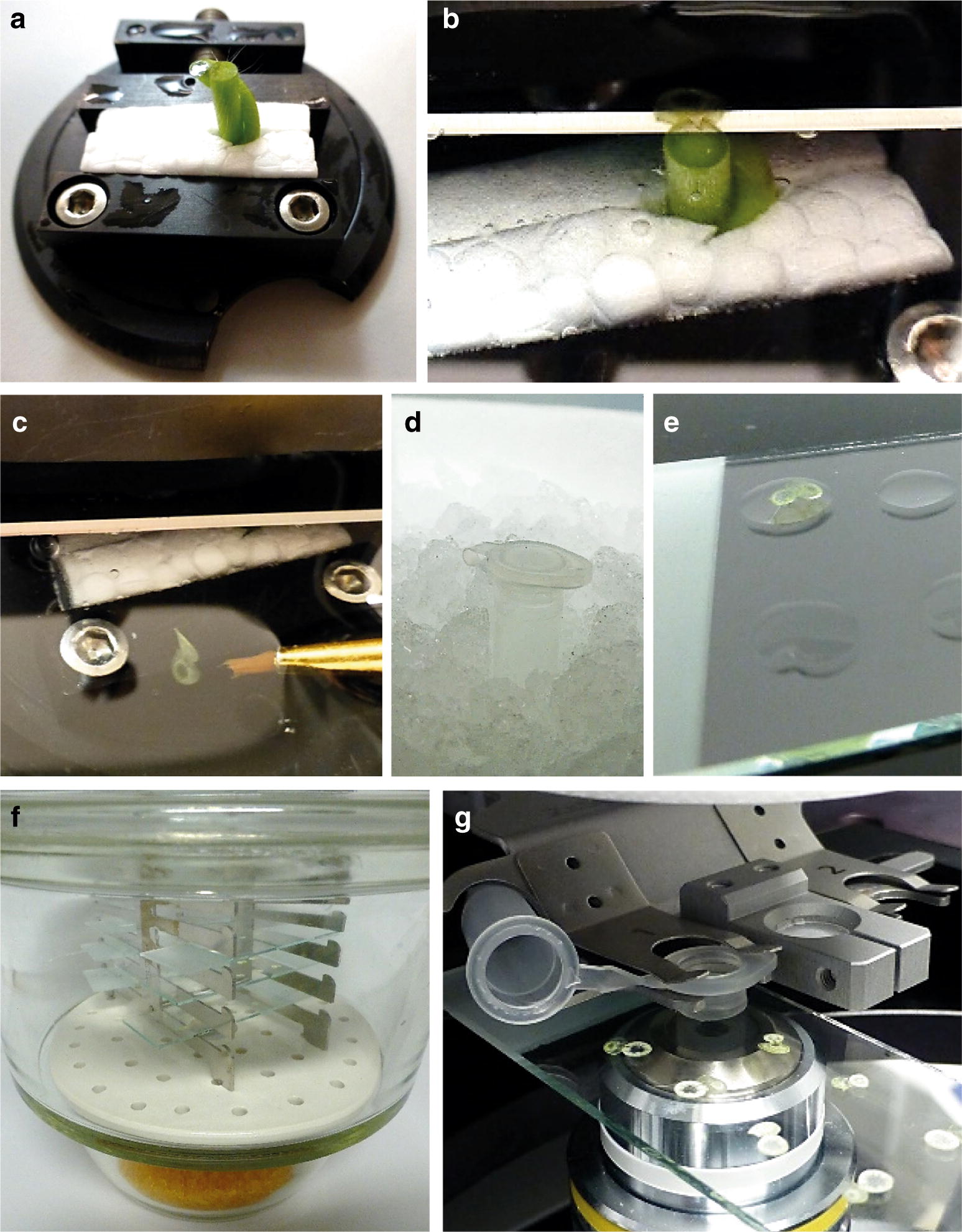
Preparation of *C. reflexa*/*P. zonale* infection sites for laser microdissection. **a** Infection sites were positioned upright in a specimen holder and **b** 100 µm cross-sections produced using a vibratome. **c** Cut sections were collected immediately and **d** stored on ice in tubes with water supplemented with RiboLock RNase Inhibitor before being **e** transferred to glass slides. After removal of excess liquid using a pipette, **f** slides with sections were dried in a desiccator at 4 °C before harvesting specific tissue regions by **g** laser microdissection

It is generally declared that protocols for preparing plant tissues for LM need to be optimized on a case-specific basis [9, 11]. Although this is true, some general factors that influence the yield and quality of RNA isolated from laser microdissected tissue have been identified [10, 12, 20]. These were considered when we developed the method for *C. reflexa* tissues. The stepwise preparation of *C. reflexa*/*P. zonale* infection sites for laser microdissection is presented in Fig. 1 and explained in detail in the Methods section. Freshly harvested infection sites were placed in a specimen holder and cut in 100 µm thick cross-sections using a vibratome (Fig. 1a, b). Takahashi et al. [20] reported that lowering the temperature at which sections are dehydrated from 42 to 4 °C, improved the integrity of isolated RNA. The same study also showed that the inclusion of an RNase inhibitor when preparing sections on slides, limited RNA degradation during longer incubation times. Accordingly, cut sections were immediately collected and kept in ice-cold water supplemented with an RNase inhibitor, before being transferred to baked non-coated glass slides and dried in a desiccator at 4 °C (Fig. 1c–f). Dryness was assessed visually and is discernible by the fact that the soft cell layers compress during dehydration, causing the section to be much thinner than the original 100 µm (Additional file 1). This procedure was sufficient to adhere the dehydrated sections to the glass slides, allowing several slides with sections ready for laser microdissection to be produced within half a working day (Fig. 1g).

### Yield and quality of RNA isolated from LM-collected plant material

**Fig. 2:**
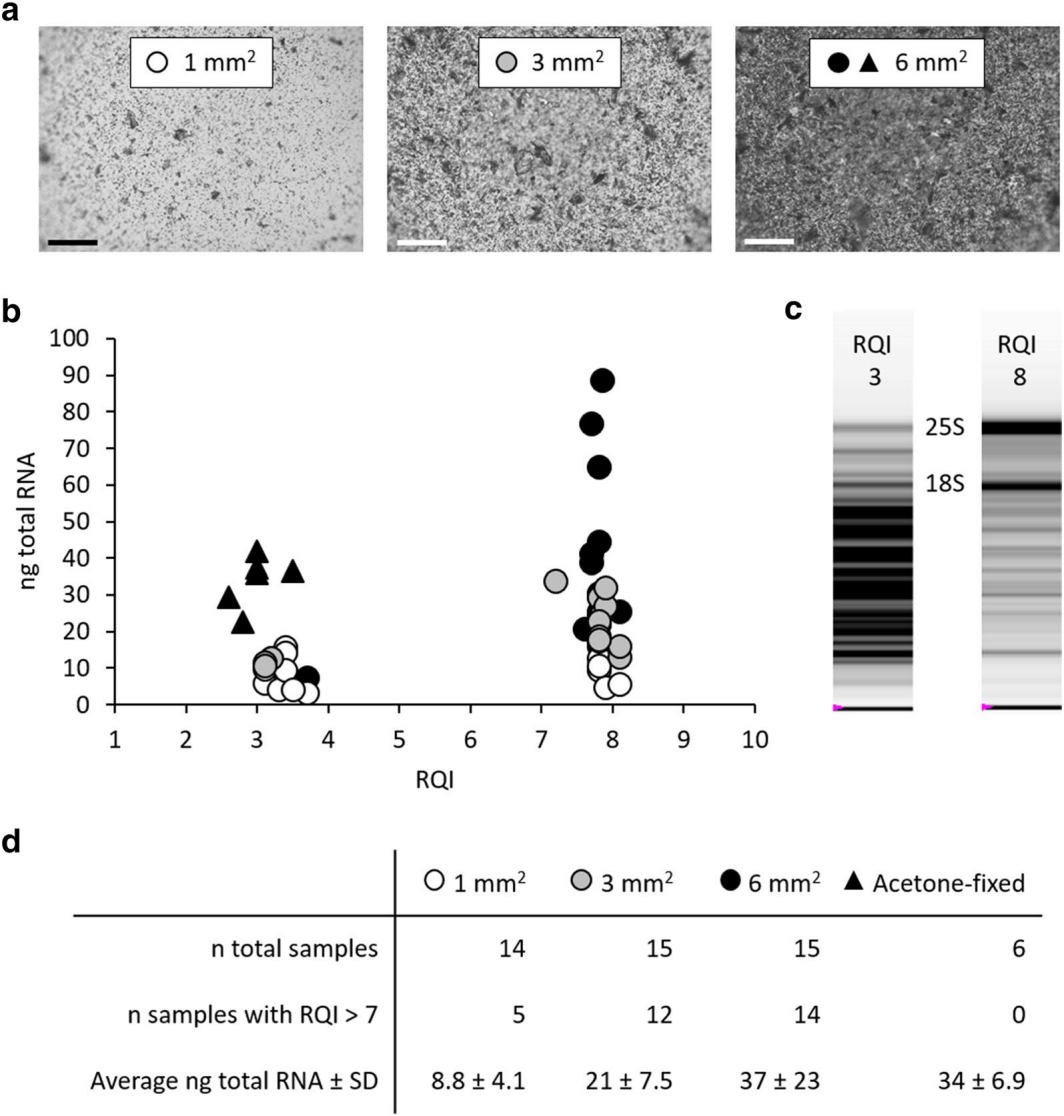
Effects of tissue quantity harvested by LM on RNA yield and integrity. **a** Surfaces of representative collection tube caps after harvest of 1 mm^2^, 3 mm^2^ and 6 mm^2^ total surface areas of *C. reflexa* tissue (scale bars = 150 µm). **b** Yields and respective RNA quality indicators (RQIs) of RNA isolated from 1 mm^2^ (white circles), 3 mm^2^ (grey circles) and 6 mm^2^ [black circles and triangles (acetone-fixed)] total surface areas. **c** Example gel pictures displaying RNA isolates with RQI = 3 and RQI = 8. **d** Average RNA yields and frequencies of samples showing high RNA quality (RQI > 7)

In order to check if this rapid preparation of plant tissues for LM was suitable for downstream gene expression analysis, RNA was isolated from three different quantities (1 mm^2^, 3 mm^2^ and 6 mm^2^ total surface areas) of *C. reflexa* tissue harvested by LM from cross-sections of *C. reflexa*/*P. zonale* infection sites (Fig. 2a). Several commercial kits have been developed specifically to facilitate the isolation of RNA from small amounts of biological material. Here, we employed the RNeasy Micro Kit (QIAGEN), which has been used previously to isolate RNA from LM-harvested plant material [15, 21, 22]. To address the success of RNA retrieval from LM-collected *C. reflexa* tissue, the quality and concentration of total RNA were measured using the Experion Automated Electrophoresis System (Bio-Rad) and the Qubit 2.0 Fluorometer (Life Technologies). The yield of total RNA had a positive linear relationship to the amount of tissue used for RNA isolation (Fig. 2d), which indicated that the isolated RNA originated from the tissue harvested by LM. RNA of high quality (RNA quality indicator (RQI) > 7) could be isolated from as little as 1 mm^2^ *C. reflexa* tissue. However, the chance of acquiring high quality RNA was generally higher when more material was used for the RNA isolation (Fig. 2). Different storage times of slides with sections in the desiccator at 4 °C prior to LM were also tested, ranging from 2 h to 2 months. Even though RNA of high quality could be isolated from 2 months old sections, the only 6 mm^2^-sample that did not yield high quality RNA, was collected from sections that had been stored for 2 months before LM. Therefore, we propose that sections prepared on slides as described above are stored in a desiccator at 4 °C for no longer than 1 month before performing LM-mediated harvest of desired regions. For all samples, the duration of storage in a desiccator at 4 °C prior to LM as well as RNA quantity and quality can be found in Additional file 2.

As mentioned, sample fixation generally stands in conflict with RNA isolation [13, 14]. However, precipitating fixatives (e.g. alcohols and acetone) do less harm to biomolecules than cross-linking fixatives [14, 23], and are commonly being used when RNA is to be isolated from laser microdissected samples. In order to test the effects of sample fixation on RNA yield and quality, 6 mm^2^ of *C. reflexa* tissue were also harvested from sections that had been fixed overnight in acetone, which has been reported to be a suitable fixative for preserving RNA through preparations for LM [10, 11, 24]. With our method, the quantitative yield of RNA was comparable between acetone-fixed and non-fixed material (Fig. 2d). However, as overnight acetone fixation appeared to reduce the quality of the isolated RNA, it was not included in the preparation procedure.

### Differential gene expression in specific tissue regions of *C. reflexa* during host infection

**Fig. 3:**
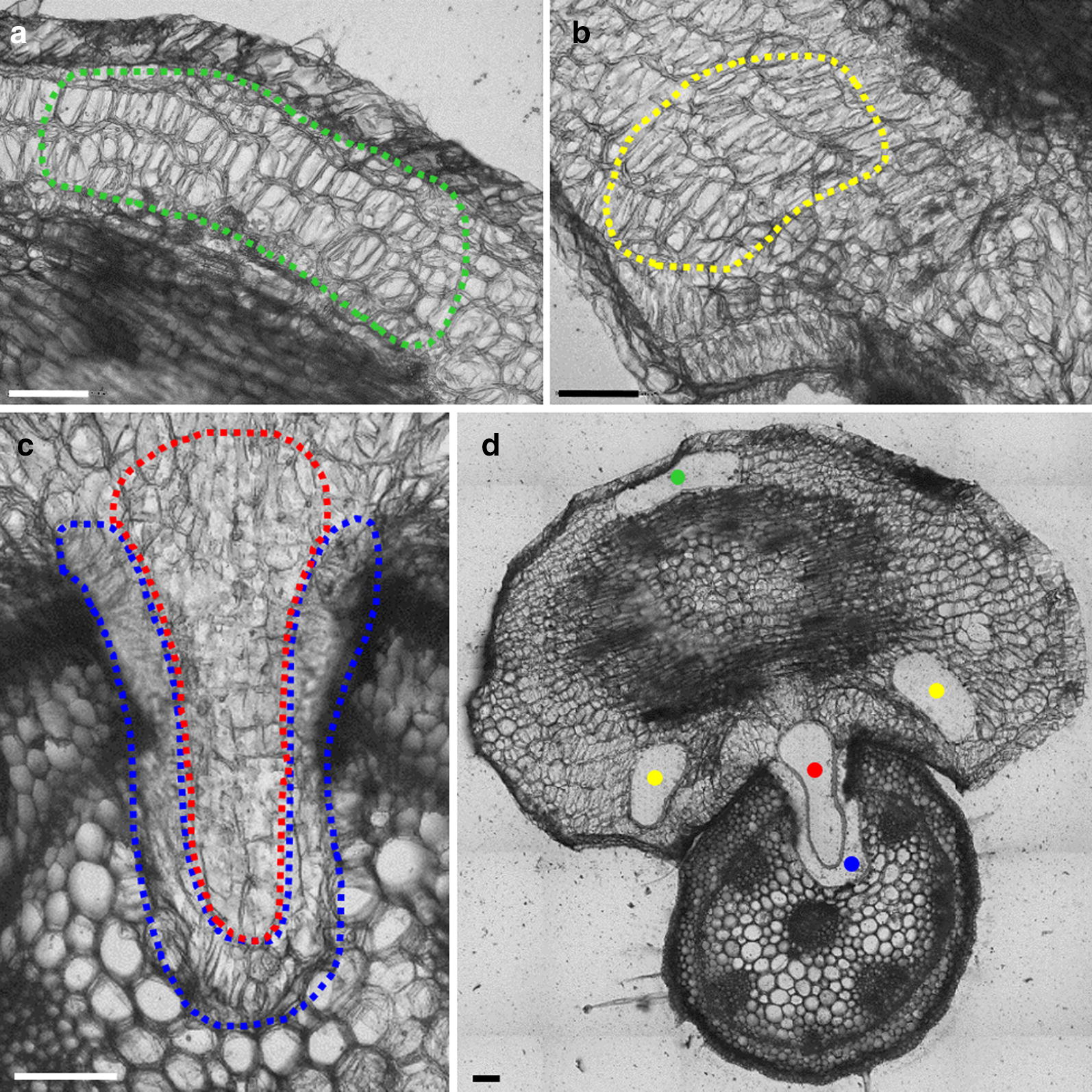
Regions of the *C. reflexa*/*P. zonale* infection site harvested by LM for gene expression analysis. **a** The parasite region of the section most distal to the host (green), **b** the swelling side regions of the parasite with elongating cells (yellow) and **c** the inner body of the haustorium (red) as well as the haustorium interface closest to *P. zonale* (blue). **d** The entire cross-section after laser microdissection. All scale bars = 150 µm

Besides the preservation of RNA quality throughout the section preparation and LM-mediated harvest of plant material, it is essential that the isolated RNA is suitable for the identification of gene-specific expression patterns. To address this, RNA was isolated from about 6 mm^2^ material collected from four different regions of the *C. reflexa*/*P. zonale* infection site (Fig. 3): the parasite region of the cross-section that is furthest away from the host plant (Fig. 3a), the side regions of the parasite containing elongating cells (Fig. 3b) as well as the inner and outer regions of the haustorium inside the host plant (Fig. 3c). Three biological replicates were collected and each replicate contained material of the respective *C. reflexa* region harvested from around 30 sections that originated from at least five different haustoria. After measuring the concentrations and qualities of RNA isolates, amplified cDNA was generated from 10 ng total RNA (RQI > 7) using the Ovation RNA-Seq System V2 (NuGEN).

**Fig. 4:**
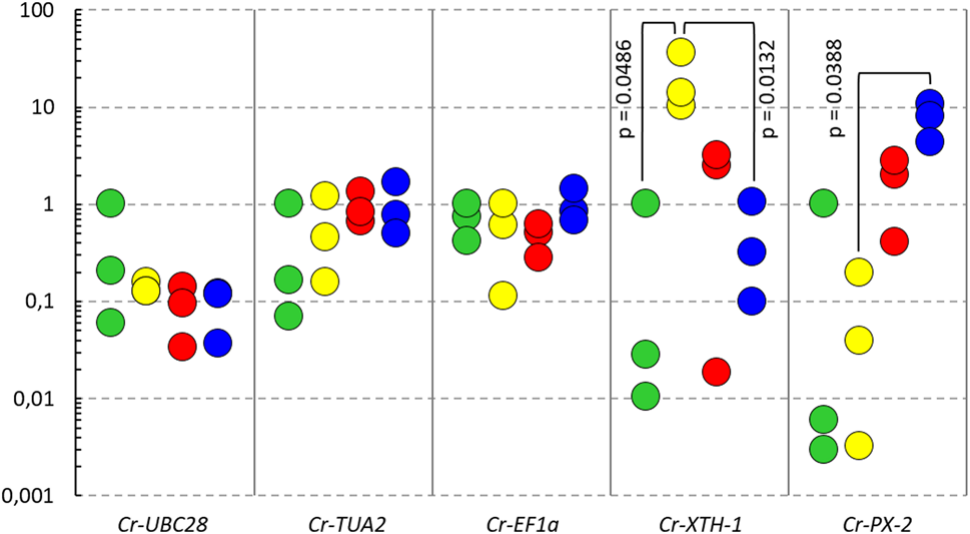
Gene expression patterns between specific regions of the host-infecting *C. reflexa.* Coloured circles indicate the normalized expression levels of respective genes in three biological replicates of the host-distal parasite region (green), the swelling side regions of the parasite (yellow), the inner haustorium (red) and the haustorium interface (blue). For each gene respectively, the biological replicate of the host-distal parasite region (green) displaying the highest normalized expression level is set to 1 and the normalized expression levels of the other samples are presented in relation. Statistically significant differences (p < 0.05) in normalized expression levels between sample types are indicated

In a previous study, we identified two cell wall-related genes, *Cr*-*PX*-*2* and *Cr*-*XTH*-*1*, which were highly expressed in young haustoria of *C. reflexa* [19]. Displaying dynamic expression patterns between larger organs of *C. reflexa* and being most highly expressed in the haustorium, these genes were good candidates to test for expressional differences between specific tissue regions of the infection organ. The expression stabilities of reference genes used to normalize expression levels across a set of samples are crucial for the generation of reliable gene expression data [25]. Thus, in addition to *Cr*-*PX*-*2* and *Cr*-*XTH*-*1*, the relative transcript quantities of five potential reference genes [*Cuscuta reflexa* ubiquitin-conjugating enzyme 28 (*Cr*-*UBC28*), *Cuscuta reflexa* tubulin alpha-2 chain (*Cr*-*TUA2*), *Cuscuta reflexa* glyceraldehyde-3-phosphate dehydrogenase C2 (*Cr*-*GAPC2*), *Cuscuta reflexa* glucose-6-phosphate dehydrogenase 6 (*Cr*-*G6PD6*) and *Cuscuta reflexa* elongation factor 1 alpha (*Cr*-*EF1a*)] were measured in the four different regions of the cross-section by performing quantitative real-time polymerase chain reaction (qPCR) on the amplified cDNA. The total span of quantification cycle (Cq) values across all samples were much smaller for the candidate reference genes than for the haustorium-related genes, *Cr*-*PX*-*2* and *Cr*-*XTH*-*1* (Additional file 3). This was expected as the candidate reference genes were selected based on earlier reports showing relatively stable expression of similarly annotated genes in other plant species [26–28]. To more precisely compare the expression stabilities of the five candidate reference genes, the NormFinder algorithm was used to calculate the stability value [29], and the coefficient of variation CV and the stability parameter M were calculated as described by Hellemans et al. [30]. Based on the calculated values (Additional file 4), *Cr*-*GAPC2* and *Cr*-*G6PD6* were chosen as the most suitable reference genes for the current sample set and normalized expression levels were calculated using the mean relative quantities of these two genes. When normalized to *Cr*-*GAPC2* and *Cr*-*G6PD6*, the expression levels of the three remaining candidate reference genes (*Cr*-*UBC28, Cr*-*TUA2* and *Cr*-*EF1a*) were more or less stable across the sample range (Fig. 4), with no differences between the tissue regions found to be statistically significant (p < 0.05). On the contrary, the normalized expression levels of the two cell wall-related genes, *Cr*-*XTH*-*1* and *Cr*-*PX*-*2*, varied much more between the investigated regions (Fig. 4). The expression of *Cr*-*XTH*-*1* was clearly highest in the swelling side regions of *C. reflexa* and were found to be significantly higher than the levels of *Cr*-*XTH*-*1*-expression in the host-distal parasite region or at the haustorium interface. The highest expression level of *Cr*-*PX*-*2* was detected at the haustorium interface and differed significantly from the expression in the swelling side regions of the parasite.

### Assessment and prevention of stress responses triggered by the preparation procedure

**Fig. 5:**
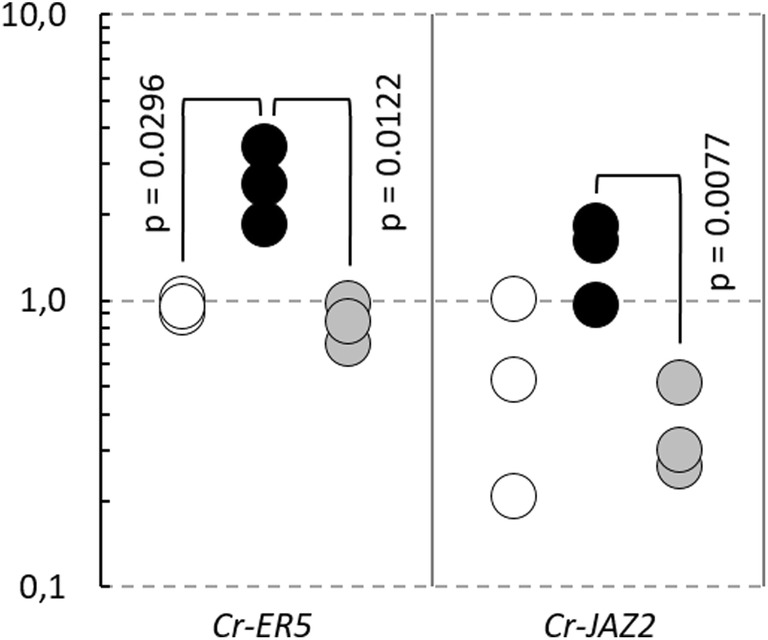
Expression of wound-related genes in differently treated *C. reflexa* tissue. Circles indicate the normalized expression levels of *Cr*-*ER5* and *Cr*-*JAZ2* in three biological replicates of *C. reflexa* material snap-frozen in liquid nitrogen (white), vibratome-sectioned *C. reflexa* material dried in a desiccator at 4 °C for at least 2 h (black) and vibratome-sectioned *C. reflexa* material dried under low pressure at 4 °C for 15 min (grey). For each gene respectively, the biological replicate of the snap-frozen *C. reflexa* material (white) displaying the highest normalized expression level is set to 1 and the normalized expression levels of the other samples are presented in relation. Statistically significant differences (p < 0.05) in normalized expression levels between sample types are indicated

The sectioning of fresh plant material followed by slow dehydration at 4 °C is prone to induce gene expression regulations in response to wounding, drought or cold stresses [31]. In tomato, the transcript abundances of the ethylene-responsive late embryogenesis-like protein 5 (*ER5*, Solyc01g095140) and the jasmonate ZIM-domain protein 2 (*JAZ2*, Solyc12g009220) have been reported to increase rapidly in response to wounding [32, 33]. The expression of *ER5* is also induced by drought [34]. To test if the LM preparation procedure described above triggers such stress responses, the expression levels of *Cr*-*ER5* and *Cr*-*JAZ2* (closest *C. reflexa* homologues to Solyc01g095140 or Solyc12g009220, respectively) were measured in parasite tissue that had been either snap-frozen in liquid nitrogen or vibratome-sectioned and dried in a desiccator at 4 °C for at least 2 h. The results show that the expression levels of both wound-related genes were higher in the vibratome-sectioned and slowly dehydrated *C. reflexa* material (Fig. 5), indicating an unwanted alteration of expression profiles. A considerable acceleration of the dehydration process was achieved by performing it under low pressure (− 0.9 bar) on a 4 °C cold metal block, shortening the required dehydration time from 2 h to 15 min. Quick drying did not influence the adhesion of the sections to the glass slides, but prevented the induction of *Cr*-*ER5* and *Cr*-*JAZ2* in the vibratome-sectioned parasite tissue (Fig. 5).

## Discussion

Laser microdissection enables the isolation of specific cell or tissue types for further downstream analysis, allowing researchers to study biological processes at a high morphological resolution. The preparatory steps up to the LM-mediated harvest of the desired tissue regions greatly affect the outcome of downstream analyses. With regards to performing gene expression analysis on RNA isolated from LM-collected material, both the quantity and the quality of the isolated RNA are of importance. Applying the method presented here, plant material can be rapidly processed from initial sample harvest to sections ready for LM. Based on the isolation of RNA from 1 mm^2^, 3 mm^2^ and 6 mm^2^ total surface areas of *C. reflexa* (Fig. 2), the average yield per mm^2^ tissue is 7 ng total RNA. Measurements of *C. reflexa* cell sizes in the sections used for LM-mediated harvest indicated that the average surface area of collected cells is 3864 µm^2^ (data not shown), which means that the average yield of total RNA per *C. reflexa* cell area is 27 pg. However, as observed by the overlapping cell walls in Fig. 3a, b, the 100 µm thick sections contain more than one cell layer in depth. Estimating that the sections encompass on average two cell layers, the average yield would be 13.5 pg total RNA per *C. reflexa* cell. This is comparable to yields reported by others isolating RNA from plant material harvested by LM [9, 24]. When harvesting material from sections that are more than one cell layer thick, the homogeneity of the microdissected material may become an issue. In the presented example, the host-invading *C. reflexa* haustorium has a uniform morphology over a stretch of 0.5–1 mm on the longitudinal axis (Additional file 5), preventing the risk of contamination with tissue types other than the ones targeted. Vibratome-produced plant tissue sections of only 20–60 µm thickness have been reported [35–38], demonstrating the possibility of applying this preparation procedure for less homogenous structures. Still, the section thickness does constitute a limitation of the presented method for use in single-cell studies.

When the laser microdissected tissue is catapulted into the adhesive cap using the AutoLPC function of the PALM MicroBeam, the collected plant material is fragmented (Fig. 2a). As the degree of tissue fragmentation is not uniform, the observed variations in RNA yield from 6 mm^2^ harvested *C. reflexa* tissue could be explained by variations in the fragment sizes of the collected material, since RNA would be extracted less efficiently from larger tissue fragments. It has also been suggested that if catapulted pieces hit areas of the adhesive cap that are already covered by LM-harvested material, they could fail to stick [21]. Even the lowest amount of total RNA isolated from harvested *C. reflexa* tissue (3 ng from 1 mm^2^, Additional file 2) would be sufficient to produce amplified cDNA using the Ovation RNA-Seq System V2 (NuGEN). As such, the described method could be applied to do gene expression analysis on much smaller amounts of LM-harvested material than the 6 mm^2^ used here. However, we observed that the probability of obtaining high quality RNA increased with the amount of collected tissue and that, when collecting 6 mm^2^, the RNA quality indicator ranged between 7.6 and 8.2 (Additional file 2). This level of RNA intactness is comparable to the highest reported integrity values for RNA isolated from LM-harvested material prepared using precipitative fixatives or by direct freezing [10, 18, 20, 39].

Among the four investigated regions of the cross-sectioned *C. reflexa*/*P. zonale* infection sites, the highest expression levels of *Cr*-*XTH*-*1* were found in the three biological replicates of the swelling side regions of the parasite. Likewise, all three biological replicates supported that the expression of *Cr*-*PX*-*2* was highest at the haustorium interface. In addition to the coinciding expression levels between biological replicates, the observed gene expression patterns make sense in light of the predicted functions of the respective gene products. *Cr*-*XTH*-*1* encodes a cell wall-modifying enzyme, the activity of which has previously been connected to plant growth in general as well as haustorium development and host plant infection in *Cuscuta* [19, 40, 41]. The high expression of *Cr*-*XTH*-*1* in the host-enveloping side regions of *C. reflexa* indicate that this enzyme could be specifically involved in the rapid elongation of cells that is occurring in this region (Figs. 3b, 4). High activity of plant peroxidases has been associated with *Cuscuta* host infection [42, 43]. The fact that the peroxidase-encoding *Cr*-*PX*-*2* gene is most highly expressed at the haustorium interface (Figs. 3c, 4), suggests that this gene product could be directly involved in plant tissue penetration or in quenching host plant defense responses. Thus, gene expression patterns identified using the presented method can provide valuable biological insight, at a high morphological resolution. When testing if stress responses were triggered by the preparation procedure, it was found that the expression levels of the wound-related genes *Cr*-*ER5* and *Cr*-*JAZ2* were higher in *C. reflexa* material dehydrated in a desiccator at 4 °C for 2 h than in snap-frozen material (Fig. 5). By performing the drying at 4 °C under low pressure, the required dehydration time was shortened to 15 min. This prevented the increased expression of both wound-related genes, implying that changes in expression levels—at least of the genes here tested—do not occur instantly. All the same, as wounding alone has been reported to induce the expression of ~ 1000 tomato genes just 30 min after treatment [33], a complete avoidance of stress gene responses cannot be expected during the processes of sectioning and drying live plant material. Still, such responses were shown to be reducible while maintaining the preparation time prior to LM at a minimum.

## Conclusions

We present a rapid procedure for the preparation of plant tissues sections for laser microdissection-mediated harvest of specific tissue regions. The method avoids fixation and embedding of plant material and utilizes a vibratome to make fresh tissue sections that are transferred to glass slides and dried at 4 °C. The short processing time allows initial sample harvest, sectioning and dehydration as well as laser microdissection, all to be carried out on the same day. RNA of high quality and sufficient quantity for gene expression analysis could be isolated from the LM-harvested material. We demonstrated the applicability of the method by identifying gene-specific expression levels in different tissue regions of the parasitic plant *C. reflexa* during infection of a host plant. Dehydration of tissue sections under low pressure reduced the drying time and prevented the induction of two stress genes.

## Methods

### Growth and harvest of plant material

*Cuscuta reflexa* was propagated on *Pelargonium zonale* under continuous light at 21 °C in a greenhouse at the Climate Laboratory of UiT The Arctic University of Norway (Holt, Tromsø). *P. zonale* petioles infected with *C. reflexa* at the penetrating stage of haustorium development (identified as described by Olsen et al. [19]) were detached from the main plant and used for sectioning.

### Sectioning and slide preparation

Freshly harvested *C. reflexa/P. zonale* infection sites were sectioned using a Leica VT1000 E vibrating blade microtome (vibratome). The apical-basal length of infection sites was trimmed to approximately 1.5 cm and positioned upright in the specimen holder, leaving about 1 cm of the infection site above the holder and accessible for sectioning. Pieces of polystyrene were placed around the specimen to ease the pressure on the plant tissue when fastening it between the clamping jaws. The specimen holder with an immobilized infection site was placed in a buffer tray that was filled with tap water to fully submerge the specimen. Sections of 100 µm thickness were made with the following settings: control knob of knife advancement speed set to 2 and control knob of knife vibration frequency set to 10. Cut sections were immediately transferred to tubes with distilled water supplemented with 1 U/µl RiboLock RNase Inhibitor (Thermo Fisher Scientific) and placed on ice. For trials with acetone fixation, cut sections were instead transferred to tubes with 100% acetone on ice. The fixative was changed once before incubation overnight at 4 °C. Single sections were transferred to 5 µl drops of distilled water supplemented with 1 U/µl RiboLock RNase Inhibitor spotted on baked (≥ 4 h at 200 °C) glass slides. Excess liquid was removed using a pipette before commencing the dehydration process. The slides with the sections were either dried in a desiccator at 4 °C for at least 2 h or dried on a 4 °C cold metal block in a vacuum chamber (− 0.9 bar) for 15 min.

### Laser microdissection

Specific regions of the infecting parasite were harvested from dry sections of *C. reflexa/P. zonale* infection sites on glass slides using the PALM MicroBeam (Zeiss) laser microdissection system with the PALMRobo V4.2 software. The circumferences of regions to be harvested were drawn using the freehand tool. Cutting and laser pressure catapulting (LPC) were done using the CloseCut + AutoLPC function with the following settings: 10× objective, 30% cut speed, 70% cut energy, 90% cut focus, 47% LPC speed, 95% LPC energy and 89% LPC focus. The selected tissue regions were catapulted into the cap of AdhesiveCap 500 clear tubes (Zeiss) placed in the TubeCollector 2 × 500 CM II (Zeiss) at a working height of − 15,356. Images were taken with the AxioCam ICc1 (Zeiss).

### RNA isolation, quality control and preparation of amplified cDNA

RNA isolation was carried out using the RNeasy Micro Kit (QIAGEN). After laser microdissection, 200 µl buffer RLT supplemented with β-mercaptoethanol (10 µl/ml) were added to the tubes with the LM-collected samples and mixed by vortexing for 30 s. Following, the tubes were kept upside down (to ensure contact of the LM-collected material in the cap and the buffer) for 5 min before vortexing again for 30 s. Tissue lysates were collected by centrifugation at 8000×*g* for 1 min and transferred to new tubes that were directly processed further or stored at − 80 °C. The remaining steps of the RNA isolation were executed following the protocol described for microdissected cryosections in the RNeasy Micro Handbook (third edition (12/2014), QIAGEN), starting from point 5 in the procedure and including on-column DNA digestion. The quantity and quality of isolated RNA were checked using the Qubit 2.0 Fluorometer with the Qubit RNA HS Assay Kit (Life Technologies) and the Experion Automated Electrophoresis System with Experion RNA HighSens Chips (Bio-Rad). The Ovation RNA-Seq System V2 (NuGEN) was used to generate amplified cDNA from total RNA. The amplified cDNA was purified using Agencourt Beads (Beckmann Coulter) as described in the Ovation RNA-Seq System V2 user guide, after which purity and concentration were measured using the NanoDrop ND-1000 Spectrophotometer (NanoDrop Technologies) and the Qubit 2.0 Fluorometer with the Qubit dsDNA HS Assay Kit (Life Technologies).

### Quantitative real-time PCR and expression analysis

Quantitative real-time polymerase chain reaction (qPCR) was set up in technical duplicates of 20 µl reactions containing 1× SsoFast EvaGreen Supermix (Bio-Rad), 1 ng purified amplified cDNA, 500 nM forward primer and 500 nM reverse primer. The CFX96 Real-Time PCR Detection System (Bio-Rad) was used for amplification and fluorescence detection with the following cycling conditions: 95 °C for 30 s followed by 40 cycles of 95 °C for 5 s and 61 °C for 5 s. After 40 cycles, melt curves of amplicons were recorded by stepwise heating from 65 to 95 °C. Data was analysed using the CFX Manager 3.1 software (Bio-Rad). All quantification cycles (Cqs) were determined by setting Single Threshold to 200 relative fluorescence units. The performance of each primer pair-specific qPCR assay was evaluated by generating standard curves using serial dilutions of purified amplified cDNA as template. Only primer pairs amplifying single qPCR products at an efficiency of 90–110% and with sizes as expected based on a *C. reflexa* transcriptome [19], were used for gene expression studies. Gene-specific primer sequences with respective amplicon sizes and qPCR amplification efficiencies, as well as melt peaks and size separation by agarose gel electrophoresis of respective qPCR amplicons are stated in Additional file 2. As some of the tissue regions of *C. reflexa* that were collected by LM are in close proximity to the host plant *P. zonale*, all qPCR primer pairs were tested on cDNA synthesized from *P. zonale* RNA in order to verify that the primers were specific to the parasite (data not shown). Relative quantities (RQs) of gene transcripts were calculated by the formula $2^{{({\text{Cq}}_{\text{control}} - {\text{Cq}}_{\text{sample}} )}}$. In samples where a gene-specific amplicon could not be detected in any of the technical duplicates, a Cq value equal to the highest measured Cq value of the respective amplicon was assigned. Expression stabilities were evaluated by calculating the coefficient of variation CV and the stability parameter M as described by Hellemans et al. [30], and by calculating the stability value using the Excel add-in “NormFinder” with Microsoft Excel 2016 [29]. Normalized expression levels were calculated by dividing the RQ of target gene against the geometric mean of the RQs of *Cr*-*GAPC2* and *Cr*-*G6PD6*, for the respective sample. The statistical significance of differences between log-transformed normalized expression levels were calculated using an unpaired two-tailed *t* test assuming unequal variances. All gene expression data and p-values are stated in Additional file 2.

## Additional files

**Additional file 1.** Vibratome-sectioned *C. reflexa/P. zonale* infection site before and after dehydration. **a** Immediately after cutting, the 100 µm thick section was placed on a glass slide that was slightly tilted in order to inspect the thickness of the sections. **b** After leaving the section to dry, the soft tissues of parasite (p) and host (h) have collapsed. Stereomicroscopy was carried out using the SteREO Lumar.V12 (Zeiss).

**Additional file 2.** RNA and qPCR data. RNA quantity, quality and duration of storage in a desiccator at 4 °C prior to LM. Gene expression data including Cq values, calculations and p-values. Gene-specific primer sequences, expected amplicon sizes and qPCR amplification efficiencies calculated based on standard curves of diluted cDNA. Size separation of qPCR amplicons on a 2% agarose gel under constant voltage set to 100 (nucleic acids were stained with GelRed and visualized using the ChemiDoc MP Imaging System (Bio-Rad). The GeneRuler 50 bp Ladder (Life Technologies) was included for size estimation). Melt peaks of qPCR amplicons generated by plotting the negative derivative of the change in fluorescence intensity as a function of temperature.

**Additional file 3.** Span of quantification cycle (Cq) values of *C. reflexa* genes. All Cq values of *Cr*-*UBC28, Cr*-*TUA2, Cr*-*GAPC2, Cr*-*G6PC2, Cr*-*EF1A, Cr*-*XTH*-*1* and *Cr*-*PX*-*2* in all 12 samples (4 tissue regions × 3 biological replicates) are included in the plot. Lower, middle and upper box lines represent the 1st quartile, 2nd quartile (median) and 3rd quartile, respectively. Whiskers show highest and lowest Cq values for the respective gene.

**Additional file 4.** Ranking of best suitable reference genes based on calculated expression stability values. The coefficient of variation CV and the stability parameter M were calculated as described by Hellemans et al. [30]. The stability value was calculated using the NormFinder algorithm [29]. For all three calculated values, lower numbers indicate more stable expression.

**Additional file 5.** Serial sectioning through a *C. reflexa* haustorium. Using a vibratome, 100 µm thick cross-sections were made in series from the start (0 mm) to the end (1.3 mm) of a single haustorium. Stereomicroscopy was carried out using the SteREO Lumar.V12 (Zeiss).

## Data Availability

The datasets supporting the conclusions of this article are included within the article and its additional files.

## Abbreviations

Cq: quantification cycle
LM: laser microdissection
LPC: laser pressure catapulting
qPCR: quantitative real-time polymerase chain reaction
RQ: relative quantity
RQI: RNA quality indicator
