# Supplementary figures and images for: A rapid preparation procedure for laser microdissection-mediated harvest of plant tissues for gene expression analysis
Source: Plant Methods. 2019 Aug 2;15:88. doi: 10.1186/s13007-019-0471-3 (PMC6676614; doi:10.1186/s13007-019-0471-3)

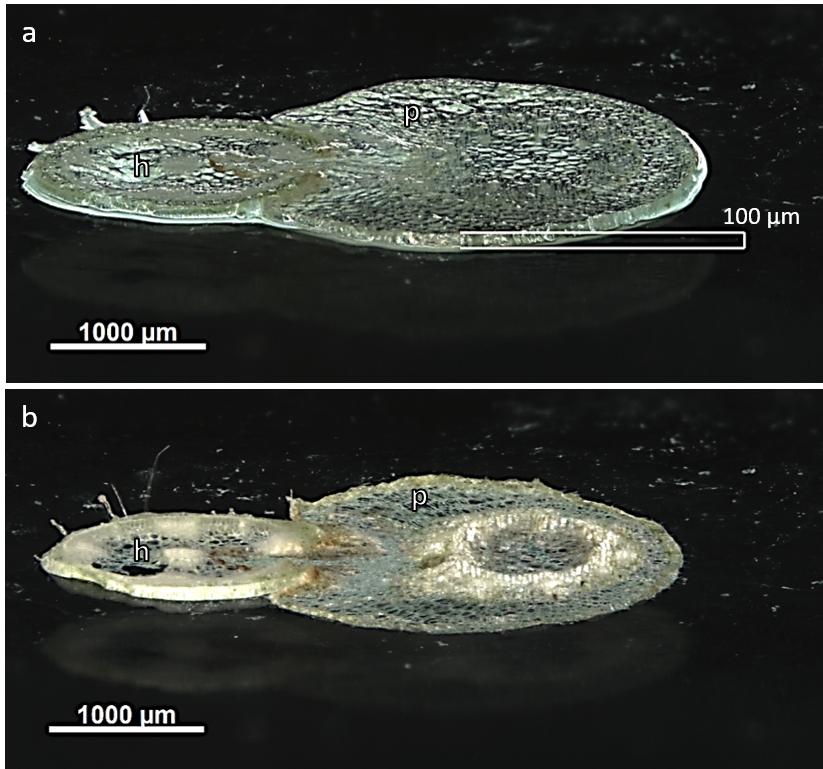

Supplement: Supplementary file 1 — Additional file 1. Vibratome-sectioned C. reflexa/P. zonale infection site before and after dehydration. a Immediately after cutting, the 100 µm thick section was placed on a glass slide that was slightly tilted in order to inspect the thickness of the sections. b After leaving the section to dry, the soft tissues of parasite (p) and host (h) have collapsed. Stereomicroscopy was carried out using the SteREO Lumar.V12 (Zeiss). [file 13007_2019_471_MOESM1_ESM.tif]

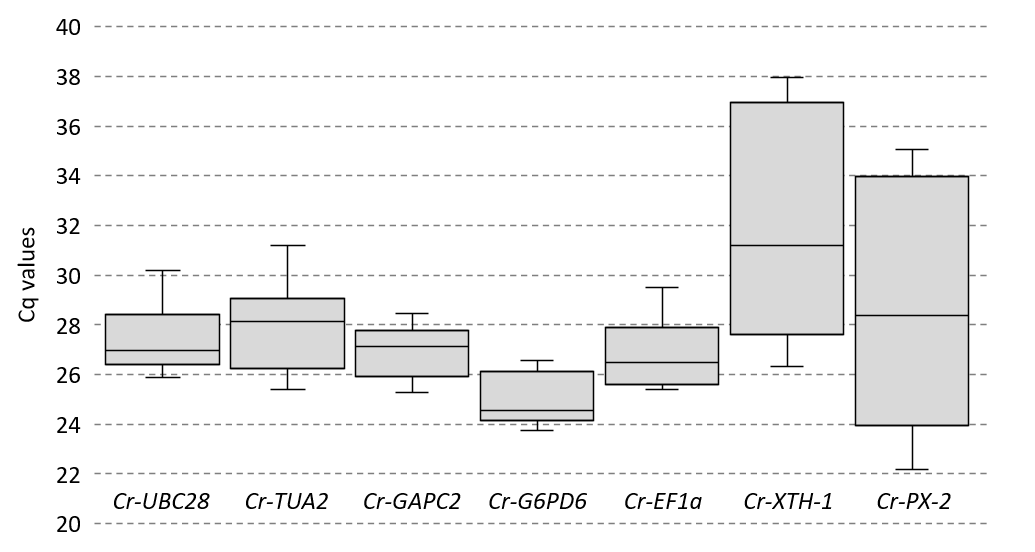

Supplement: Supplementary file 3 — Additional file 3. Span of quantification cycle (Cq) values of C. reflexa genes. All Cq values of Cr-UBC28, Cr-TUA2, Cr-GAPC2, Cr-G6PC2, Cr-EF1A, Cr-XTH-1 and Cr-PX-2 in all 12 samples (4 tissue regions × 3 biological replicates) are included in the plot. Lower, middle and upper box lines represent the 1st quartile, 2nd quartile (median) and 3rd quartile, respectively. Whiskers show highest and lowest Cq values for the respective gene. [file 13007_2019_471_MOESM3_ESM.tif]

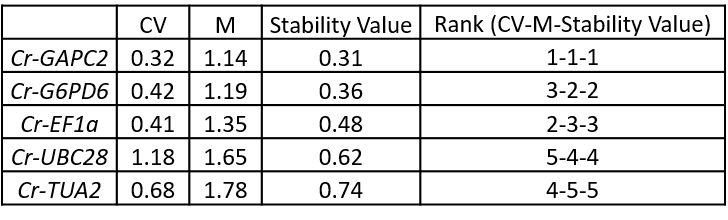

Supplement: Supplementary file 4 — Additional file 4. Ranking of best suitable reference genes based on calculated expression stability values. The coefficient of variation CV and the stability parameter M were calculated as described by Hellemans et al. [30]. The stability value was calculated using the NormFinder algorithm [29]. For all three calculated values, lower numbers indicate more stable expression. [file 13007_2019_471_MOESM4_ESM.tif]

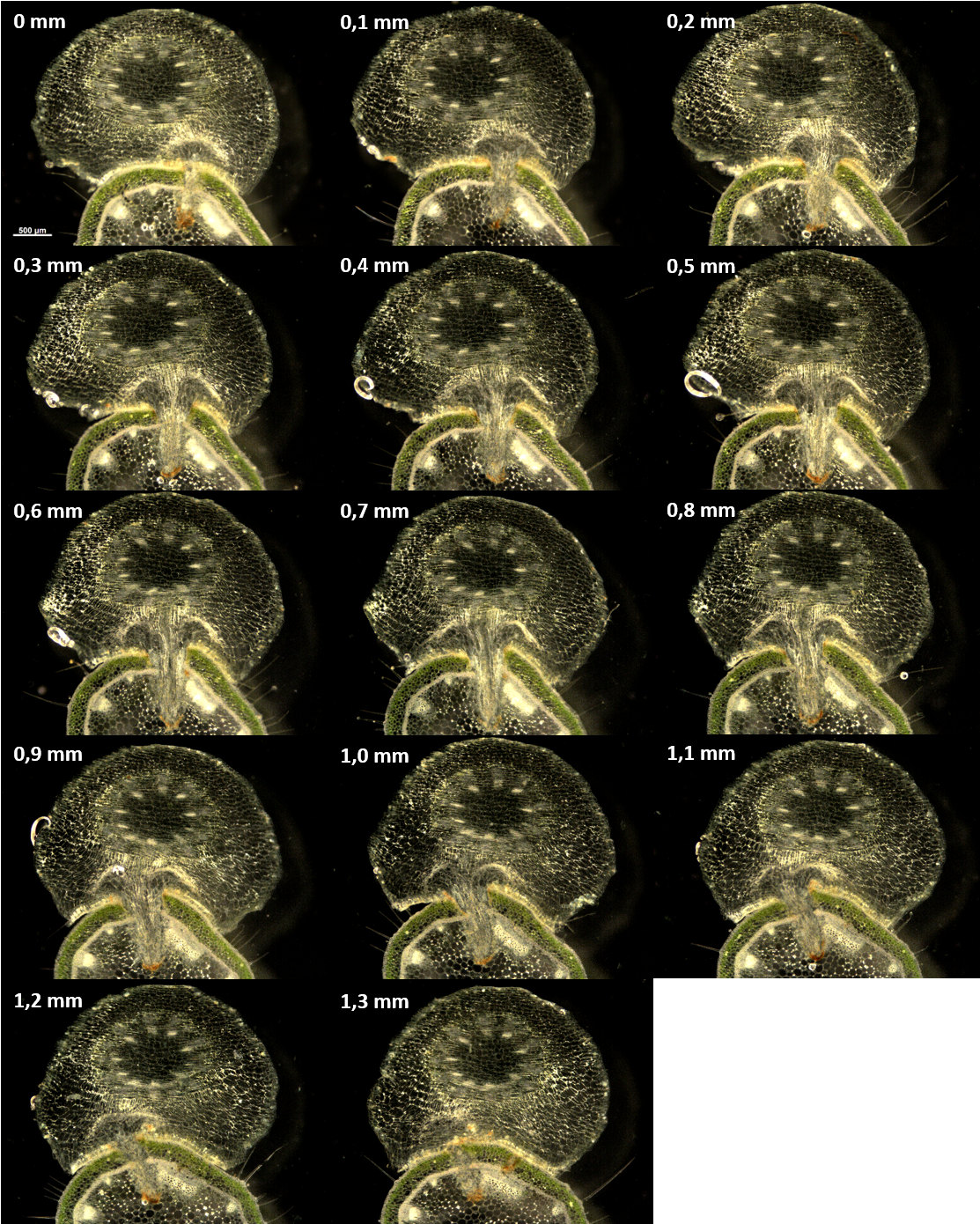

Supplement: Supplementary file 5 — Additional file 5. Serial sectioning through a C. reflexa haustorium. Using a vibratome, 100 µm thick cross-sections were made in series from the start (0 mm) to the end (1.3 mm) of a single haustorium. Stereomicroscopy was carried out using the SteREO Lumar.V12 (Zeiss). [file 13007_2019_471_MOESM5_ESM.tif]
